# Examining the Utilization of Social Capital by Ghanaians When Seeking Care for Chronic Diseases: A Personal Network Survey

**DOI:** 10.3389/ijph.2023.1605891

**Published:** 2023-12-21

**Authors:** Brady Hooley, Elom Hillary Otchi, Samuel Mayeden, Alfred Edwin Yawson, Koku Awoonor-Williams, Fabrizio Tediosi

**Affiliations:** ^1^ Swiss Tropical and Public Health Institute (Swiss TPH), Basel, Switzerland; ^2^ University of Basel, Basel, Switzerland; ^3^ Korle Bu Teaching Hospital, Accra, Ghana; ^4^ Accra College of Medicine, Accra, Ghana; ^5^ Ghana Health Service, Accra, Ghana; ^6^ Medical School, University of Ghana, Accra, Ghana; ^7^ Department of Policy, Planning, Monitoring and Evaluation, Ghana Health Service, Accra, Ghana

**Keywords:** Ghana, NCDs, social capital, social support, informal care

## Abstract

**Objectives:** With limited social security and health protection in Ghana, intergenerational support is needed by those living with NCDs, who incur recurrent costs when seeking NCD care. We measured the level of informal support received by NCD patients and identified factors that influence support provision.

**Methods:** We surveyed 339 NCD patients from three hospitals in Ghana, who listed their social ties and answered questions about their relationship and support frequency. We analyzed the relationship between social support, demographic and health information, characteristics of social ties, and network characteristics.

**Results:** Participants described 1,371 social ties. Nearly 60% of respondents reported difficulties in their usual work or household duties due to chronic illness, which was also the strongest predictor of support. Patients with higher wellbeing reported less social support, while older age and having co-habitant supporters were negatively associated with support, indicating caregiver burnout.

**Conclusion:** Ghanaian NCD patients receive support from various caregivers who may not be able to handle the increasing healthcare and social needs of an aging population. Policies should therefore enhance resource pooling and inclusiveness for old age security.

## Introduction

Ghana is facing a growing challenge of both communicable and non-communicable diseases [1, 2]. Limited availability of health services for NCDs has made it difficult for patients to receive the care they need, complicating their engagement in care [3]. The current system, as outlined in the Ghana Health Service (GHS) Community-based Health Planning and Services (CHPS) policy, limits certain types of healthcare professionals and facilities from providing some services, which leads to inadequate and unequal access to NCD care services, particularly at the primary care level [1].

In Ghana, primary healthcare services are delivered by a mix of public, faith-based, and self-financing private facilities, organized into a multi-level system consisting of district hospitals, health centers, and CHPS Compounds, providing basic public health and clinical services [4]. Although some form of NCD care is available within each level of the health system, medications for NCDs are not regularly offered at health centers and CHPS Compounds, which are the most accessible facilities for the majority of the population [5]. CHPS Compounds may provide NCD screening services such as blood pressure and blood glucose measurement. However, these facilities must refer patients to higher levels of the health system for definitive NCD diagnoses and treatment. The NCD services provided at health centers can be more variable and depend on the specific credentials of the facility, yet a shortage of healthcare professionals adequately trained to provide NCD care at CHPS Compounds and health centers drives patients to self-refer to the district hospital level (or higher) for even basic NCD care services [6].

For rural populations in particular, substantial opportunity costs associated with travelling to seek care can further drive this already vulnerable group into poverty and hinder their engagement in care and adherence to treatment [6, 7]. While most patients attending public health facilities have health insurance to mitigate the direct medical costs of obtaining NCD care, patients often rely upon financial support from their family and friends to cope with the non-medical costs of care-seeking. However, this support tends to wane throughout the duration of one’s chronic illness [8–10].

Intergenerational support is a form of social capital and a cultural norm in sub-Saharan Africa (SSA), whereby parents support and care for their children and in turn, their children support them as they age [11]. Social capital is considered a sympathetic behavior that extends beyond a reciprocal or transactional relationship [12], compensating for a lack of widespread social security for aging adults in sub-Saharan Africa, and plays a major role in pooling resources and facilitating older patients’ access to healthcare services [13]. In this manner, intergenerational support is grounded on principles of sympathy, reciprocity, and a sense of responsibility and duty. However, as the population ages and the burden of communicable and non-communicable diseases increases, the burden of providing support also increases and undermines the reliability of the support that the older patients can expect to receive [11, 14].

The receipt of financial support may be particularly important for people living with NCDs, who experience substantial recurrent travel and opportunity costs when accessing centralized NCD care services [15]. Previous research directly surveyed caregivers in Ghana to study the financial burden of caring for older family members and found that the purchase of household goods represented the largest direct cost, with direct transfers of money being relatively less prevalent [16]. This study investigated the personal networks of patients seeking care for chronic diseases in Ghana. Employing multilevel models, the study aimed to identify factors that influence their mobilization of social capital. Additionally, the results revealed the cumulative cost of support borne by the patients’ social networks.

## Methods

This study used a personal network survey to quantify NCD patients’ receipt of informal support in relation to their socioeconomic and health status, and personal network characteristics. Similar to classic “whole” network data, personal network data allows one to study a network of social ties in relation to individual members of a key population; in this case, patients seeking care for NCDs [17–19].

We aimed to recruit 100 NCD patients each from the Tamale Teaching Hospital, Kintampo North Municipal Hospital, and Hohoe Municipal Hospital, located in the Northern, Middle, and Southern zones of Ghana, respectively. As patients seeking care for NCDs frequently experience difficulties in receiving appropriate care from community-level health facilities [6], we purposively selected hospitals in order to facilitate the recruitment of patients with chronic illnesses. In March and April 2022, trained research assistants recruited potential respondents as they waited to be seen by a clinician and administered the questionnaire in English or a local language following their consultation. Patients were eligible for participation if they had ever been diagnosed with at least one chronic health condition, were at least 50 years of age, and did not display any indications of cognitive impairment. We also collected three blood pressure measurements from each participant, such that we report the mean of the final two measurements. Data was collected using tablets and Open Data Kit (ODK) [20], and data was uploaded to a secure server in Switzerland at the end of each day of data collection.

### Questionnaire

The questionnaire, described in greater detail in the Supplementary Material, is similar to that used in a previous study [21]. We first collected information on participants’ sociodemographic information and chronic disease history before asking participants to list the six most important adults in their lives and provide information on the informal social support provided by these members of their social network (Supplementary Box S1).

The key outcome of interest was how frequently named social ties provide support to their respective participants, and the questionnaire asked participants to report on their social networks’ provision of emotional, informational, and material support, described to participants as:

- Emotional support: “How frequently does [a given social tie] give you emotional support? Such as comforting you, making you feel respected or loved, or praying with/for you.”
- Informational support: “How frequently does [a given social tie] give you informational support? Such as sharing advice and knowledge, or helping you understand your doctor’s instructions.”
- Material support: “How frequently does [a given social tie] give you material support? Such as giving you money for healthcare or bus tickets, helping you with tasks at home, taking you to the health facility.”

### Analysis

Our primary aim was to assess the amount of material and non-material support received as an outcome, including support from individual alters and the cumulative support experienced by participants. In our primary analysis, we combined emotional and informational support into a single category of non-material support. In our Supplementary Material we also provide separate analyses for emotional support and informational support, allowing for a more comprehensive examination of the data.

We quantified social support using a categorical measure of frequency, which we converted to a count of person-days of support per month (as seen in Supplementary Table S1) [21, 22]. We used descriptive statistics to explore participant characteristics and participant-level summaries of network and support characteristics, and bivariate analyses to explore predictors of support provision. For alter-level variables we used linear regression while clustering variance at the participant level and for participant-level variables we used Welch t-tests.

We initially planned to employ Poisson regression to investigate the relationship between our predictor variables and the count of person-days of support exchanged between individuals (alters) and our study participants. However, due to the observed overdispersion of the outcome data, we found it necessary to utilize negative binomial regression, a determination supported by likelihood ratio tests [23, 24]. Similar to Poisson regression, negative binomial regression yields incidence rate ratios. These ratios indicate that, for categorical predictor variables, the incidence rate at one category level is x times that of the reference level. For continuous predictor variables, the incidence rate ratio demonstrates the impact of a one-unit increase, representing a 1-*x* percent difference in the outcome.

To investigate the influence of various participant-level, tie-level, and network-level predictors on social support in terms of the count of person-days of support provided over the past month, we employed multilevel negative binomial regression. Additionally, we included a random intercept term to account for participant-level clustering. Our modelling process unfolded in four incremental steps: we constructed separate models for material and non-material support. We gradually integrated the participants' random intercept, participant-level predictors, tie-level predictors, and network-level predictors to create the comprehensive model.

At the participant level, we investigated if participants’ age, Warwick-Edinburgh Mental Wellbeing Scale (WEMWBS) score [25], Multidimensional Scale of Perceived Social Support (MSPSS) score, chronic illness-related productivity limitations, and multimorbidities were associated with the receipt of support. The WEBWBS is a validated self-report scale designed to measure an individual’s mental wellbeing or psychological flourishing. The abridged version consists of 7 items covering aspects such as positive affect, satisfying interpersonal relationships, and a sense of personal accomplishment. Respondents rate their experiences over the past 2 weeks on a 5-point Likert scale. The WEMWBS is widely used in research and clinical settings to assess an individual’s mental wellbeing and to monitor changes in mental health and wellbeing over time [25]. Likewise, the MSPSS is also a validated self-report scale and uses 12 items rated on a 7-point Likert scale to measure an individual’s perceived social support [26–28]. For both the WEMWBS and MSPSS, scores of the individual items are summed to arrive at a single score, which were included in the models as continuous predictor variables. However, due to excessive collinearity, we did not include the MSPSS in the final model specifications.

At the alter level, we sought to determine if family members and household members differ in their support provision relative to non-family ties or those living outside the household.

In terms of network characteristics, we hypothesized that ties would provide less support to participants who have a larger number of supportive ties overall while participants with more non-family ties would receive more support. The rationale underlying these hypotheses is that a “bystander effect” may lead individual ties to be less likely to support the participant if they perceive there to be others who are willing to provide support, while non-family ties and those not residing in the same household may be more willing and able to support the participant if they are less likely to have experienced caregiver fatigue.

We used STATA version 16 for data cleaning and manipulation, R version 4.2.1 for analyses, and Python 3.9.7 and the “NetworkX” package for network visualization.

### Ethics Statement

This study received ethical approval from the Korle Bu Teaching Hospital (KBTH) Institutional Review Board (IRB) (Ref: KBTH-STC 000147/2021). Prior to recruitment, we presented the study’s objectives to potential participants and described in detail the information they would be asked to provide. We informed potential participants that they may refuse blood pressure measurements and/or withdraw from the study at any time without consequences and all participants provided written informed consent before participation. In cases where potential participants were unable to write, we accepted verbal consent *in lieu* of written consent.

## Results

This study interviewed a total of 339 participants, who provided information about their relationships with 1,371 alters. Patients had a mean age of 62.4 years, most were married or living with a partner (65.2%), just over half had completed primary school, and only 23.6% had a formal occupation. Diabetes (44.2%) and hypertension (69%) were the most commonly-reported chronic diseases, and nearly 60% of respondents reported being unable to accomplish their usual work or household duties due to their chronic illness (Table 1). On average, participants named four social ties each, of which 80% provided emotional support, 40% provided informational support, and 50% provided financial or other material support (Table 1).

**TABLE 1:** Summary of participant-level variables disaggregated by gender, with chi-square *p*-values (Ghana, March and April 2022).

		Overall	Women	Men	*p*-value
*n*		339	173	166	
Age, mean (SD)		62.4 (10.1)	62.5 (10.3)	62.3 (9.9)	0.859
Marital status, *n* (%)	Divorced	19 (5.6)	10 (5.8)	9 (5.4)	<0.001***
	Widowed	63 (18.6)	55 (31.8)	8 (4.8)	
	Never Married	9 (2.7)	2 (1.2)	7 (4.2)	
	Living with partner	26 (7.7)	7 (4.0)	19 (11.4)	
	Married	195 (57.5)	81 (46.8)	114 (68.7)	
	Separated	27 (8.0)	18 (10.4)	9 (5.4)	
Education, *n* (%)	None	106 (36.3)	64 (40.3)	42 (31.6)	0.017*
	Some primary	44 (15.1)	28 (17.6)	16 (12.0)	
	Primary	31 (10.6)	17 (10.7)	14 (10.5)	
	Some secondary	34 (11.6)	21 (13.2)	13 (9.8)	
	Secondary	41 (14.0)	13 (8.2)	28 (21.1)	
	College	36 (12.3)	16 (10.1)	20 (15.0)	
Household size, mean (SD)		5.5 (3.6)	5.1 (2.9)	5.9 (4.1)	0.025*
Occupation, *n* (%)	Caring for home/children	25 (8.7)	22 (13.6)	3 (2.4)	<0.001***
	Private Formal Sector	17 (5.9)	4 (2.5)	13 (10.3)	
	Public Servant	51 (17.7)	20 (12.3)	31 (24.6)	
	Retired	54 (18.8)	21 (13.0)	33 (26.2)	
	Self-employed, small business	94 (32.6)	71 (43.8)	23 (18.3)	
	Subsistance Farmer	47 (16.3)	24 (14.8)	23 (18.3)	
Paid work in past year, *n* (%)	Yes	117 (34.5)	55 (31.8)	62 (37.3)	0.336
Productivity loss due to illness, *n* (%)	Never	147 (43.4)	71 (41.0)	76 (45.8)	0.533
	Sometimes	99 (29.2)	55 (31.8)	44 (26.5)	
	Completely	93 (27.4)	47 (27.2)	46 (27.7)	
Days of work/productivity lost to illness, mean (SD)		10.4 (13.2)	10.5 (13.0)	10.4 (13.4)	0.954
Current HI, *n* (%)	Yes	282 (83.4)	147 (85.5)	135 (81.3)	0.380
Paid for own HI, *n* (%)	I do not know	1 (0.4)	1 (0.7)		0.002**
	No	19 (6.7)	14 (9.5)	5 (3.7)	
	Yes, partially	17 (6.0)	15 (10.2)	2 (1.5)	
	Yes, completely	245 (86.9)	117 (79.6)	128 (94.8)	
Perceived health status, *n* (%)	Bad	35 (10.9)	20 (12.3)	15 (9.4)	0.039*
	Moderate	110 (34.3)	66 (40.7)	44 (27.7)	
	Good	151 (47.0)	65 (40.1)	86 (54.1)	
	Very good	25 (7.8)	11 (6.8)	14 (8.8)	
Diabetes, *n* (%)	Yes	150 (44.2)	87 (50.3)	63 (38.0)	0.029*
Hypertension, *n* (%)	Yes	234 (69.0)	121 (69.9)	113 (68.1)	0.799
Epilepsy, *n* (%)	Yes	9 (2.7)	3 (1.7)	6 (3.6)	0.328
Asthma, *n* (%)	Yes	14 (4.1)	9 (5.2)	5 (3.0)	0.459
Other chronic illness, *n* (%)	Yes	36 (10.6)	12 (6.9)	24 (14.5)	0.038*
Systolic BP, mean (SD)		139.3 (19.6)	139.1 (20.9)	139.5 (18.3)	0.881
Diastolic BP, mean (SD)		92.1 (16.5)	92.5 (17.4)	91.8 (15.5)	0.713
Stage II Hypertension, n (%)	Yes	218 (64.3)	116 (67.1)	102 (61.4)	0.335
Tie count, mean (SD)		3.9 (1.9)	3.9 (1.9)	4.0 (1.9)	0.849
Tie weight, mean (SD)		0.7 (0.1)	0.8 (0.1)	0.7 (0.1)	0.016*
Proportion of women in support network, mean (SD)		0.4 (0.3)	0.4 (0.3)	0.4 (0.3)	0.760
% of alters who provide emotional support, mean (SD)		0.8 (0.3)	0.9 (0.3)	0.8 (0.3)	0.381
% of alters who provide informational support, mean (SD)		0.4 (0.4)	0.4 (0.4)	0.3 (0.4)	0.076
% of alters who provide material support, mean (SD)		0.5 (0.4)	0.5 (0.4)	0.4 (0.4)	0.010*

* = *p* < 0.05, ** = *p* < 0.01, *** = *p* < 0.001.

**FIGURE 1:**
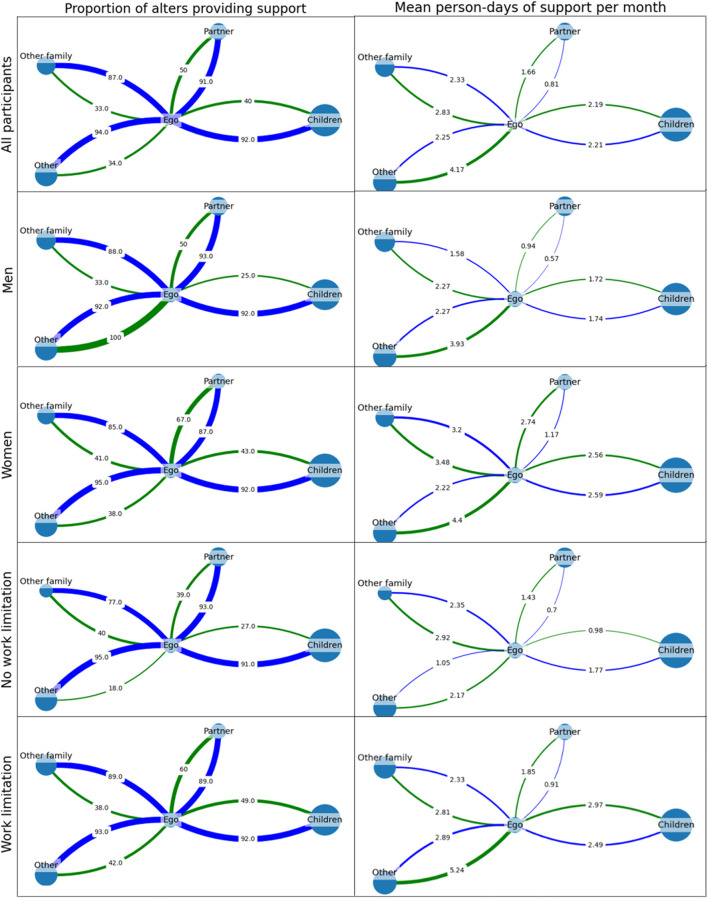
Average cluster graphs depicting participants’ receipt of support from each of four classes of social tie. Node size corresponds to the relative prevalence of each relation type within the network. The left column represents the proportion of ties that provide non-material support (blue) and material support (green), while the right represents the average number of support events provided by each class of tie per month (Ghana, March and April 2022).

Initial exploratory analyses using bivariate statistics suggested that men receive less non-material support (6.7 person-days) compared to women (9.9 person-days) and less material support (8.9 person-days) compared to women (12.7 person-days) (Supplementary Table S and Figure 1). Figure 1 demonstrates these between-group differences, both in terms of the proportion of one’s social ties that provide support and the frequency of support received by patients. However, when controlling for other variables in multivariate mixed effects negative binomial regression models, we did not find a significant difference in the amount of support received by men and women (Tables 2, 3).

**TABLE 2:** Multilevel negative binomial regression models for predicting the count of person-days of informal social support provided by alters to egos over the past month (*N*_2_ = 339, *N*_1_ = 1,371) (Ghana, March and April 2022).

Support type:	Overall		Non-material		Material	
Predictors	Incidence Rate Ratios	*p*	Incidence Rate Ratios	*p*	Incidence Rate Ratios	*p*
(Intercept)	5.63*** (3.53–8.98)	<0.001	3.22*** (2.06–5.05)	<0.001	3.56*** (1.99–6.38)	<0.001
Ego age	0.80** (0.70–0.92)	0.001	0.81*** (0.71–0.91)	<0.001	0.88 (0.74–1.03)	0.119
Ego gender: Men	0.90 (0.70–1.16)	0.426	0.86 (0.68–1.08)	0.195	0.90 (0.65–1.24)	0.510
Living with partner: Yes	0.81 (0.61–1.08)	0.158	0.96 (0.74–1.25)	0.788	0.67* (0.47–0.96)	0.028
Wellbeing score	0.86* (0.75–0.98)	0.026	0.80*** (0.71–0.91)	<0.001	1.02 (0.87–1.20)	0.765
Only hypertension: Yes	0.76 (0.55–1.05)	0.097	0.94 (0.70–1.26)	0.687	0.68 (0.45–1.01)	0.058
Multimorbidities: Yes	0.89 (0.65–1.23)	0.487	0.90 (0.68–1.19)	0.455	0.92 (0.62–1.36)	0.662
Ever missed work due to NCD: Yes	2.63*** (1.96–3.53)	<0.001	1.90*** (1.45–2.48)	<0.001	3.46*** (2.39–5.02)	<0.001
Alter relation: partner	1.06 (0.76–1.47)	0.728	0.48** (0.29–0.80)	0.004	1.54* (1.03–2.29)	0.034
Alter relation: Other family	0.78* (0.62–0.99)	0.042	0.85 (0.65–1.10)	0.211	0.56*** (0.40–0.78)	0.001
Alter relation: other	0.84 (0.67–1.04)	0.108	0.96 (0.74–1.24)	0.740	0.56*** (0.41–0.75)	<0.001
Alter same age or older than ego: Yes	0.90 (0.73–1.11)	0.321	0.84 (0.66–1.07)	0.156	1.05 (0.78–1.41)	0.761
Alter gender: Men	1.43*** (1.22–1.68)	<0.001	1.18 (0.98–1.42)	0.074	1.49*** (1.21–1.85)	<0.001
Alter resides with ego: yes	0.23*** (0.19–0.28)	<0.001	0.17*** (0.14–0.22)	<0.001	0.26*** (0.20–0.34)	<0.001
% of “other” ties composing network	1.08 (0.97–1.21)	0.153	0.97 (0.88–1.08)	0.620	1.18* (1.03–1.35)	0.019
Number of named social ties	0.89** (0.82–0.96)	0.004	0.89** (0.82–0.96)	0.004	0.86** (0.77–0.95)	0.004

*p < 0.05 **p < 0.01 ***p < 0.001.

**TABLE 3:** Negative binomial regression models for predicting the total count of person-days of informal social support received by egos from all of their alters over the past month (*N* = 339) (Ghana, March and April 2022).

Support type:	Non-material		Material	
Predictors	Incidence Rate Ratios	*p*	Incidence Rate Ratios	*p*
(Intercept)	35.18*** (10.23–120.96)	<0.001	21.00*** (5.44–81.05)	<0.001
Ego age	0.98* (0.97–1.00)	0.013	0.99 (0.98–1.00)	0.109
Ego gender: Men	0.88 (0.68–1.15)	0.354	0.80 (0.59–1.07)	0.138
Living with partner: Yes	0.86 (0.64–1.16)	0.323	0.70* (0.51–0.97)	0.033
Wellbeing score	0.93*** (0.90–0.95)	<0.001	0.95*** (0.93–0.98)	0.001
Hypertension: Yes	0.88 (0.57–1.36)	0.570	0.87 (0.54–1.39)	0.557
Diabetes: Yes	1.21 (0.78–1.88)	0.383	1.11 (0.69–1.80)	0.672
Ever missed work due to NCD: Yes	1.90*** (1.36–2.65)	<0.001	2.59*** (1.80–3.73)	<0.001
% of “other” ties composing network	1.37 (0.83–2.25)	0.213	1.78* (1.07–2.96)	0.027
Number of named social ties	1.20*** (1.11–1.29)	<0.001	1.14** (1.05–1.24)	0.002
% of women composing network	0.97 (0.61–1.53)	0.880	1.18 (0.73–1.92)	0.507
Hypertension: Yes × Diabetes: Yes	0.89 (0.51–1.57)	0.696	0.99 (0.54–1.82)	0.973

*p < 0.05 **p < 0.01 ***p < 0.001.

The provision of non-material support was influenced by several factors, including the age of the patient, WEMWBS score, productivity loss due to chronic illness, and number of reported social ties. Additionally, the relationship between the patient and their social ties, the gender of the social ties, and their residence location also played a significant role in determining the level of non-material support provided (Table 2, see Supplementary Table S3 for disaggregated analysis of emotional and informational support). In terms of household and gender, negative binomial regression models indicated that egos’ partners and those living in the same household provided less support, while men tended to provide more support than women (Table 2).

We also found that alters provided more support to younger egos, those with work limitations, and lower WEMWBS scores, yet provided less support to those with a larger network of social ties (Table 2, Figure 1). Figure 1 clearly demonstrates the differences in the amount of support received by people with and without NCD-related work limitations, particularly in how “other” social ties provide substantially more support to people living with work limitations than without. Together, the directionalities of these effects suggest that alters provide the most support to patients with the greatest need for support. Among the predictors at the social ties level, partners and those living in the same household provided the least amount of support, suggesting compassion fatigue.

We analyzed the provision of tie-level support and found that WEMWBS score, chronic illness-related productivity loss, and number of reported social ties were important predictors of overall non-material support received by the ego (Table 3, see Supplementary Table S4 for disaggregated analysis of emotional and informational support). This pattern was also observed when analyzing overall material support received, with additional factors such as living with a partner and the proportion of non-family ties in one’s social network also playing a role (Table 3).

For the provision of material support, we found that egos’ marital status, chronic illness-related productivity loss, number of non-kin social ties, and overall number of reported social ties were important predictors, while alters’ relation to the ego, gender, and residence location were also important predictors (Table 2). As with non-material support, alters provided more support to egos whose chronic condition limits their ability to work. However, they provided notably less support to those who were married and had a larger network of social ties (Table 2). While there was no association between the proportion of non-family ties in egos’ networks and the provision of non-material support, egos with more non-family ties receive significantly more material support than those whose networks were mostly composed of family members. However, non-family ties and “other” family ties provided less support than egos’ children or partners (Table 3), suggesting that the overall structure and composition of social networks is as important as the characteristics of individual social ties when it comes to determining the mobilization of social capital.

## Discussion

This study examined the personal networks of patients to understand the factors that drive the mobilization of social capital among Ghanaians seeking care for NCDs. We found important predictors of support mobilization at the individual, social network, and relationship levels. These predictors contributed to significant variability in both giving and receiving social support, yielding some surprising findings. Contrary to our expectations, we observed that older patients and those with better mental wellbeing did not receive the most support, despite older patients’ greater need for it and the demonstrated protective effect of receiving support, respectively [29].

However, the relationship between mental wellbeing and social support is complex and may involve multiple potential causal pathways to consider. While previous research has shown that social support can have a protective effect on mental wellbeing [29], it is also possible that individuals with higher levels of mental wellbeing are perceived as having less need for support. Further investigation is needed to fully understand the different patient typologies that may exist. A larger, longitudinal study would be beneficial, as it would allow for the examination of the dynamic relationships between mental wellbeing and social support over time, and establish the temporal precedence of these variables.

Our study found that older patients generally need more support, but surprisingly, they receive less support from their social network. This could be due to caregiver fatigue and burnout, or the reduction of social connections over time [30, 31]. The occurrence of compassion fatigue and burnout is further supported by our finding that alters living in the same household as the ego provide substantially less non-material and material support, and that one’s partner also provides less non-material support than other types of relation (Table 2, Supplementary Table S2).

The strongest predictor of social support with the largest effect size was whether patients’ chronic illness ever prevented them from working or performing their usual household duties. Patients’ reduced ability to work is also a more readily observed outcome of their illness and the greater provision of support to these patients may demonstrate the need-based provision of support or even the redistribution of household or family resources to account for variable, differential labor productivity.

Contrary to previous work in Ghana and sub-Saharan Africa [16, 32], we found that care recipients more frequently reported men as caregivers than women.

Previous studies focused on caregiving from the perspective of caregivers, which may explain unexpected differences in care recipients’ perception and reporting of informal care. For example, gender roles and expectations (e.g., women providing informal care and performing household work) could cause underreporting of women’s contributions to care [33]. To have a more complete understanding of the burden and function of informal care, future studies should involve both caregivers and care recipients.

Although participants more frequently identified family members as social ties, non-family ties were reported to provide more support during the 1 month recall period. Furthermore, having a higher proportion of non-family ties within one’s network was predictive of receiving greater overall support. On an individual basis, non-family ties may provide more support over this relatively short 1 month recall period if, for example, they are less routinely mobilized relative to family ties.

Previous research has investigated the notion that “weaker” non-family ties provide bridging social capital, which expands the patient’s pool of resources and enables the sharing and exchange of both financial and non-financial support [34–36]. Bridging social capital can give patients access to new resources, reducing the risk of resource depletion through overuse. These ties, being less familiar, may be less prone to compassion fatigue compared to stronger ties, such as close relatives.

The conclusions drawn from this study bear limitations, primarily due to its exclusive focus on patients who are seeking healthcare, without comparing them to a random sample of people with NCDs. The challenge of obtaining such a sample via a household survey is underscored by the low awareness of hypertension among Ghanaians, with estimates as low as 20% [37–39]. Additionally, it is important to note that the results may not fully represent the true extent of social support experienced by NCD patients, as they rely solely on self-reports from the patients themselves. For instance, there is a possibility that respondents may have underreported support received from household members, as they may perceive such assistance as routine family or household resource-sharing, thus not explicitly categorized as “social support.” Conversely, receiving support less frequently from individuals outside the household may be seen as a more exceptional event, making it easier to recall and report as social support. To obtain a more holistic understanding of informal support for NCD patients, future research should consider adopting a mixed-methods approach. This approach should involve both the recipients and providers of support, facilitating an exploration of the motivations, determinants, and functions of support, while also addressing any disparity in perceptions between the two groups.

### Conclusion

Patients seeking care for NCDs reported receiving support from a variety of caregivers, and we identified a number of factors that influence the level of support received. As the population in SSA ages and the burden of chronic illness increases, the demand for informal care may soon outstrip the ability of younger generations to provide it. To address this issue, policymakers should focus on improving resource pooling and inclusivity for old age security and social health protection, reducing the financial stress of aging and chronic illness for both caregivers and recipients. Additionally, they should consider expanding old age security, while respecting traditional customs of intergenerational support, by aligning social security schemes and poverty reduction strategies with national values and expectations.
