# Supplementary material for: Examining the Utilization of Social Capital by Ghanaians When Seeking Care for Chronic Diseases: A Personal Network Survey
Source: Int J Public Health. 2023 Dec 21;68:1605891. doi: 10.3389/ijph.2023.1605891 (PMC10764420; doi:10.3389/ijph.2023.1605891)
Supplement: Supplementary file 1 [file DataSheet1.pdf]

Supplementary Materials

Box S1: Questionnaire details

The questionnaire, described in greater detail in a previous study (Hooley, Mtenga, and Tediosi 2022), consisted of several distinct thematic sections:

1. Sociodemographics,
2. Social health protection,
3. Chronic disease history and engagement in care,
4. The Warwick-Edinburgh Mental Well-being Scale (WEMWBS) (Tennant et al. 2007),
    a. Abridged version of WEMWBS, featuring seven positively-phrased 5-point Likert scale questions, such as “I’ve been feeling relaxed,” and I’ve been feeling interested in new things.” The scale is scored out of 35, with scores above 27.5 being indicative of “high wellbeing.” While the scale has not be specifically validated in a Ghanaian population, however its construct and cross-cultural validity has been validated in various other sub-Saharan African contexts, including Niger, Kenya and Tanzania.
5. The multidimensional scale of perceived social support (MSPSS) {Wilson, 2017 #762;Zimet, 1988 #559}.
    a. 12, 7-point Likert scale questions such as “I get the emotional help and support I need from my family,” and “I can count on my friends when things go wrong.” The MSPSS is scored out of 84, with scores between 12 and 35 indicating low perceived support, scores between 36 and 60 indicating medium perceived support, and scores between 61 and 84 indicating high perceived support. The MSPSS has been validated in a wide range of cultural contexts, including Ghana.
6. ‘Name generator’ (Bidart & Charbonneau 2011; Fernández-Peña, Molina, and Valero 2018; McCarty et al. 2019b),
7. ‘Name interpreter’

Sections one through five collected information on the participants themselves (the egos) while sections six and seven collected information on the characteristics and support behaviour of members of their social network (the alters).

The “name generator” asked participants to (Bidart & Charbonneau 2011; Fernández-Peña, Molina, and Valero 2018; McCarty et al. 2019b):

‘[…] list up to 6 people in your social environment. List people that you have talked to either in person or by phone or internet, at least one time in the past 6 months. List the people who are the most important to you for any reason, and indicate the type of relationship that you have with this person. This list can include people such as your family members, friends, neighbours, or elders.’

The ‘name interpreter’ stage of the questionnaire asked participants a loop of questions for each alter that was elicited by the name generator. These questions asked about the attributes of each alter, such as their age, gender and residential proximity to the ego.

We then specifically asked egos about the frequency of contact with the alter and the support that they may receive from the alter. We began by generically asking participants, ‘has (alter) supported you in coping with your chronic disease in the past 6 months? ‘Support’ may include emotional support, prayer, food, time, labour, money or sharing helpful knowledge and information’. Subsequent questions then gathered more information on the following three sub-types of support:

- Emotional support, such as providing comfort to the ego, making them feel respected or loved, or praying with/for them
- Informational support, such as sharing advice and knowledge, or helping to understand doctor's instructions
- Material support, such as giving money for healthcare or bus fare, providing transport to the health facility, or cooking and helping with other tasks at home

For the purposes of analysis, emotional support and informational support were pooled together as non-material support.

For alters who provided material support, we asked more detailed information about the type of material support and the amount of support given in the case that an alter gave money to an ego.

Table S1: Categorical variable used to quantify the provision of social support (left), and the categories' corresponding conversion to a count variable (right).

Social support frequency, as collected	Social support frequency, as analysed (person-days per month)
Monthly	1
A few times a month	2
Weekly	4
A few times a week	10
Daily/almost daily	30

Table S2: Mean differences in number of social support events between levels of ego-level and alter-level predictor variables. Significance testing used unequal variances t-tests for predictor variables at the ego level, and linear regressions adjusted for ego-level clustering for predictor variables at the alter level.

		Non-material support events per month			Material support events per month		
Variable	Variable level	Mean (SE)	95% CI	p-value	Mean (SE)	95% CI	p-value
Ego gender	Women (n=173)	9.87 (1.11)	7.67-12.08	0.0072**	12.65 (1.39)	9.90-15.40	0.0235*
	Men (n=166)	6.69 (0.65)	5.41-7.97		8.93 (1.24)	6.48-11.38	
Ego age	< 60 years (n=164)	8.75 (0.92)	6.94-10.56	0.2599	11.79268 1.410332	9.007806 14.57756	0.1605
	> 60 years (n=175)	7.90 (0.94)	6.04-9.76		9.92 1.249773	7.453333 12.38667	
Marital status	Widowed (n=118)	9.19 (1.19)	6.82 11.55	0.1743	14.05932 1.676861	10.73839 17.38026	0.0073**
	Living with a partner (n=221)	7.85 (.78)	6.30 9.39		9.099548 1.113802	6.90446 11.29464	
NCD affects ability to work	No (n=147)	4.93 (0.91)	3.14 6.72	<0.001***	4.959184 1.121285	2.743136 7.175231	<0.001***
	Yes (n=192)	10.90 (0.89)	9.15 12.65		15.31771 1.332484	12.68943 17.94598	
Multimorbidities	No (n=224)	7.59 (0.74)	6.14 9.04	0.0773	9.53125 1.092053	7.379185 11.68331	0.0331*
	Yes (n=115)	9.72 (1.30)	7.15 12.29		13.34783 1.754369	9.872435 16.82322	
Stage II Hypertension	No (n=121)	9.18 (1.01)	7.17 11.19	0.1544	14.19835 1.853357	10.52883 17.86787	0.0070**
	Yes (n=218)	7.83 (0.85)	6.15 9.51		8.954128 1.017888	6.947916 10.96034	
Alter-level variables		Mean difference (SE)	95% CI	p-value	Mean difference (SE)	95% CI	p-value
Alter gender	Women (n=522)	ref					

	Men (n=849)	.488587 .2441757	.0082916 .9688824	0.046*	.6321094 .3549196	-.06602 1.330239	0.076
Alter gender x ego gender interaction	Woman x woman (n=292)	ref					
	Woman x man (n=230)	-.7862716 .3074893	-1.391105 -.1814379	0.011*	-.8600357 .5341341	-1.910681 .19061	0.108
	Man x woman (n=405)	.6043886 .4113295	-.2046996 1.413477	0.143	.8170979 .547837	-.2605015 1.894697	0.137
	Man x man (n=444)	-.2794952 .3505456	-.9690211 .4100306	0.426	-.2612304 .5592876	-1.361353 .8388925	0.641
Alter age	Younger than ego (n=1160)	ref					
	Same age or older than ego (n=211)	-.255965 .2982971	-.8427176 .3307876	0.391	.2250572 .5586147	-.8737419 1.323856	0.687
Daily contact with alter	No (n=522)	ref					
	Yes (n=849)	-3.653697 .3409115	-4.324272 -2.983121	<0.001***	-3.661759 .3895117	-4.427931 -2.895587	<0.001***
Relation type	Child (n=661)	ref					
	Partner (n=177)	-1.399352 .2282631	-1.848347 -.9503569	<0.001***	-.5322273 .4342439	-1.386388 .3219337	0.221
	Other family (n=218)	.1230135 .3964771	-.6568598 .9028869	0.757	.6426807 .5446887	-.428726 1.714087	0.239
	Other (n=315)	.0403573 .3466702	-.6415456 .7222602	0.907	1.983834 .5579972	.8862495 3.081419	<0.001***
Alter resides in same household	No (n=622)	ref					
	Yes (n=749)	-2.879619 .3078783	-3.485218 -2.27402	<0.001***	-2.857435 .3442745	-3.534625 -2.180244	<0.001***

Table S3: Multilevel negative binomial regression models for predicting the count of person-days of emotional and informational support provided by alters to egos over the past month (N_2=339, N_1=1371)

	**Emotional support**		**Informational support**	
Predictors	*Incidence Rate Ratios*	*p*	*Incidence Rate Ratios*	*p*
(Intercept)	2.26 ** (1.37 – 3.74)	**0.001**	1.79 (0.99 – 3.23)	0.053
ego age cen	0.87 * (0.77 – 0.99)	**0.035**	0.73 *** (0.61 – 0.87)	**0.001**
ego sex [1]	0.76 * (0.59 – 0.98)	**0.034**	0.93 (0.67 – 1.28)	0.650
livingpartner [1]	1.00 (0.76 – 1.33)	0.977	1.03 (0.71 – 1.48)	0.884
wemwbs cen	0.76 *** (0.67 – 0.87)	**<0.001**	0.95 (0.80 – 1.13)	0.591
hypertension only [1]	1.04 (0.76 – 1.44)	0.794	0.82 (0.55 – 1.23)	0.333
multimorbidity [1]	0.95 (0.70 – 1.29)	0.755	0.80 (0.54 – 1.19)	0.272
preventwork ever [1]	1.39 * (1.04 – 1.86)	**0.026**	2.80 *** (1.91 – 4.12)	**<0.001**
reltype 2 [2]	0.57 (0.28 – 1.17)	0.128	0.45 ** (0.25 – 0.81)	**0.008**
reltype 2 [3]	1.00 (0.74 – 1.34)	0.990	0.61 * (0.39 – 0.93)	**0.022**
reltype 2 [4]	1.16 (0.86 – 1.56)	0.329	0.62 * (0.42 – 0.92)	**0.016**
alter same age [Yes]	0.89 (0.68 – 1.17)	0.418	0.76 (0.52 – 1.11)	0.157
alter sex [1]	1.18 (0.95 – 1.47)	0.133	0.95 (0.73 – 1.24)	0.724
samehh [1]	0.07 *** (0.05 – 0.10)	**<0.001**	0.39 *** (0.29 – 0.52)	**<0.001**
other tie cen	0.89 (0.79 – 1.00)	0.056	1.16 * (1.01 – 1.34)	**0.042**
tiecount	0.94 (0.86 – 1.02)	0.128	0.72 *** (0.65 – 0.80)	**<0.001**

** p<0.05   ** p<0.01   *** p<0.001*

Table S4: Negative binomial regression models for predicting the total count of person-days of informal social support received by egos from all of their alters over the past month (N=339)

	Emotional support		Informational support	
Predictors	*Incidence Rate Ratios*	*p*	*Incidence Rate Ratios*	*p*
(Intercept)	26.30 *** (6.87 – 100.66)	**<0.001**	6.43 * (1.28 – 32.44)	**0.024**
ego age	0.98 * (0.97 – 1.00)	**0.022**	0.98 * (0.96 – 1.00)	**0.012**
ego sex [1]	0.81 (0.61 – 1.07)	0.143	0.86 (0.62 – 1.18)	0.345
livingpartner [1]	0.93 (0.67 – 1.29)	0.673	0.98 (0.68 – 1.40)	0.894
wemwbs	0.91 *** (0.89 – 0.94)	**<0.001**	0.98 (0.95 – 1.01)	0.136
hypertension [1]	0.96 (0.59 – 1.56)	0.863	0.71 (0.42 – 1.20)	0.198
diabetes [1]	1.42 (0.87 – 2.30)	0.158	1.03 (0.60 – 1.76)	0.914
preventwork ever [1]	1.71 ** (1.20 – 2.44)	**0.003**	2.89 *** (1.91 – 4.35)	**<0.001**
other tie	1.44 (0.84 – 2.47)	0.189	1.72 (0.91 – 3.26)	0.095
tiecount	1.25 *** (1.15 – 1.37)	**<0.001**	1.01 (0.92 – 1.12)	0.799
prop women	0.91 (0.54 – 1.52)	0.712	1.22 (0.70 – 2.12)	0.490
hypertension [1] × diabetes [1]	0.88 (0.48 – 1.63)	0.693	0.97 (0.47 – 1.99)	0.932

** p<0.05   ** p<0.01   *** p<0.001*
